# Efficient Gene Targeting by Homologous Recombination in Rat Embryonic Stem Cells

**DOI:** 10.1371/journal.pone.0014225

**Published:** 2010-12-03

**Authors:** Stephen Meek, Mia Buehr, Linda Sutherland, Alison Thomson, John J. Mullins, Andrew J. Smith, Tom Burdon

**Affiliations:** 1 The Roslin Institute and Royal (Dick) School of Veterinary Studies, University of Edinburgh, Roslin, Midlothian, Scotland, United Kingdom; 2 Molecular Physiology Laboratory, Centre for Cardiovascular Science, Queen's Medical Research Institute, University of Edinburgh, Edinburgh, Scotland, United Kingdom; 3 MRC Centre for Regenerative Medicine, Institute for Stem Cell Research, School of Biological Sciences, University of Edinburgh, Edinburgh, Scotland, United Kingdom; Brigham and Women's Hospital, United States of America

## Abstract

The rat is the preferred experimental animal in many biological studies. With the recent derivation of authentic rat embryonic stem (ES) cells it is now feasible to apply state-of-the art genetic engineering in this species using homologous recombination. To establish whether rat ES cells are amenable to *in vivo* recombination, we tested targeted disruption of the hypoxanthine phosphoribosyltransferase (*hprt*) locus in ES cells derived from both inbred and outbred strains of rats. Targeting vectors that replace exons 7 and 8 of the *hprt* gene with neomycinR/thymidine kinase selection cassettes were electroporated into male Fisher F344 and Sprague Dawley rat ES cells. Approximately 2% of the G418 resistant colonies also tolerated selection with 6-thioguanine, indicating inactivation of the *hprt* gene. PCR and Southern blot analysis confirmed correct site-specific targeting of the *hprt* locus in these clones. Embryoid body and monolayer differentiation of targeted cell lines established that they retained differentiation potential following targeting and selection. This report demonstrates that gene modification via homologous recombination in rat ES cells is efficient, and should facilitate implementation of targeted, genetic manipulation in the rat.

## Introduction

The rat was first domesticated for scientific research over 100 years ago and rapidly became one of the most important experimental animal models in biomedical sciences [1]. Its size, physiology, intelligence and reproductive characteristics make it a particularly useful model to study most facets of mammalian biology, including human disease. Despite these advantages, progress in applying forward genetic approaches to dissect the genetic and molecular basis of biological processes in rats has languished behind the rapid advances made in mice, particularly those made through applying homologous recombination in embryonic stem (ES) cells. A limiting step in applying this technology to rats has been the lack of genuine germ line competent rat ES cells. However, a novel serum-free culture system using small molecule differentiation inhibitors was recently shown to support the derivation and propagation of genuine rat ES cell lines [2], [3]. These cell lines can be transmitted through the germ line and provide an opportunity to apply contemporary in-vivo DNA recombination based methods to deliver targeted genetic engineering in the rat.

To evaluate the potential of these novel rat ES cell lines for introducing targeted mutations in the rat, we have tested their capacity for homologous recombination at the *hprt* locus. The hprt enzyme catalyses a key step in the scavenger pathway for purine synthesis and its inactivation can be selected for directly, either positively or negatively, by chemically manipulating nucleotide biosynthesis. The gene encoding HPRT is located on the X-chromosome and was amongst the first genes to be successfully targeted by homologous recombination in mouse, in an attempt to model the mutation that causes Lesch-Nyhan syndrome in humans [4], [5].

Manipulation of the *hprt* gene also has direct applications in genetic engineering [6], [7], [8], [9]. The *hprt* locus, with its ubiquitous, low level, constitutive transcriptional activity can be exploited as a “safe haven” for expressing exogenous transgenes [10]. Targeted integration of transgenes within the *hprt* locus, using, for example, recombination mediated cassette exchange [11], [12], permits both comparative analysis of genes placed at the identical genomic site, as well as tight experimental control of conditionally regulated transgenes [13], [14], [15]. In addition, *hprt*-deficient ES cells provide a host background in which recombination-mediated reconstruction of *hprt* minigenes can be used in chromosome engineering [8], [9], [16].

In this report we demonstrate efficient homologous recombination at the *hprt* locus in ES cells derived from inbred and outbred strains of rats. We compared the targeting efficiencies in these lines with those previously obtained with ES cells of other species, and evaluated the differentiation potential of correctly targeted clones, to assess the feasibility of gene targeting in the rat using ES cells.

## Results and Discussion

**Figure 1:**
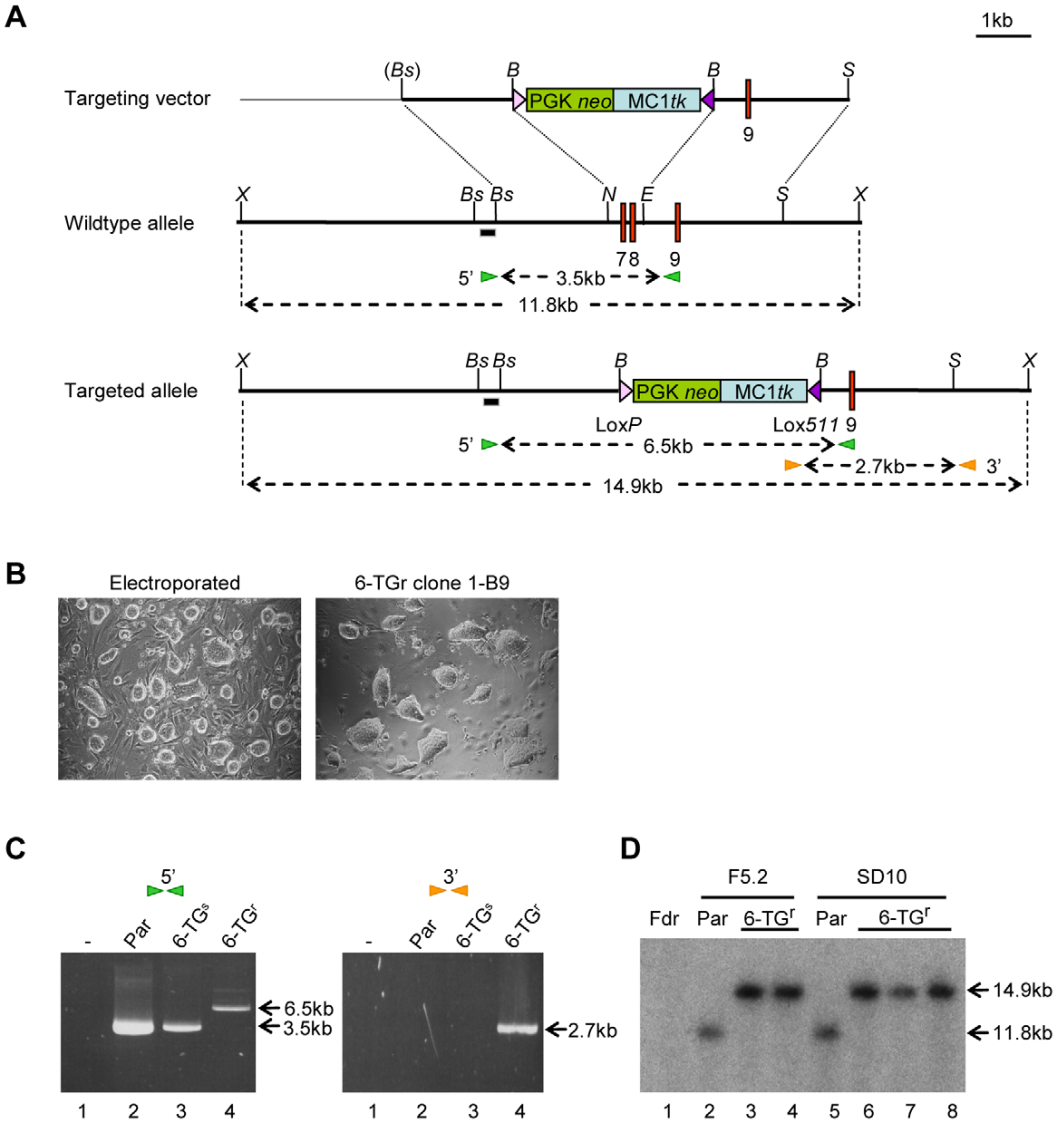
Targeting of the *hprt* gene in rat embryonic stem cells. (A) Structure of the HPRT targeting vector (top), the wild-type *hprt* allele (middle) and targeted allele (bottom), resulting from replacement recombination at the dotted lines. The null allele was created by substitution of exons 7 and 8 with a PGK*neo*/*MC1tk* selection cassette (green and blue boxes). Exons are depicted by red boxes, non-exon–containing chromosomal, and cloned, genomic DNA sequence is shown by a thick black line and pBluescript plasmid sequence by a thin black line. Restriction enzyme sites BamHI (*B*) BstBI (*Bs*), *Eco*RV (*E*), *Nde*I (*N*), *Sac*I (*S*) and *Xba*I (*X*) are indicated. Oligonucleotide pairs (green and orange arrowheads) and 5′ probe sequence (hashed box), consisting of sequence homologous and external to the homology arms, were used for PCR-based and Southern screening respectively. Sizes of expected products are shown by dotted arrows. (B) Brightfield image of electroporated RIF5.2 cells two days post-electroporation and prior to selection (left panel), and of a resultant 6-TG-resistant clone 1-B9 (right panel) (Magnification x100). (C) Confirmation of targeted integration by PCR amplification of (1) water blank, and of genomic DNA from (2) RIF5.2 parental rat ES cell line, (3) 6-TG-sensitive wildtype clone and (4) 6-TG-resistant targeted RIF5.2 clone using oligonucleotide pairs shown in (A). (D) Confirmation of targeted integration by Southern blot analysis using 5′ probe shown in panel (A), of XbaI digested genomic DNA from (1) SNL feeder cells, (2) RIF5.2 parental rat ES cell line, (3) RIF5.2-derived 6-TG-resistant clone 1-B9, (4) RIF5.2-derived 6-TG-resistant clone 3-B4, (5) RISD10 parental cell line, (6) RISD10-derived 6-TG-resistant targeted clone 13, (7) RISD10-derived 6-TG-resistant targeted clone 14 and (8) RISD10-derived 6-TG-resistant targeted clone 16.

Based on previous reports describing targeted disruption of the *hprt* gene in mouse and human ES cells, the *hprt* targeting vector was designed to delete exons 7 and 8 of the rat gene, thereby ensuring its complete inactivation (Figure 1). A 7 kb fragment spanning this region was amplified from Fischer 344 (F344) rat genomic DNA by PCR, using oligonucleotide primers based on genomic sequence information available for the Brown Norway (BN) strain. Sub-fragments of this amplicon, flanking exons 7 and 8, provided the 5′ and 3′ homology arms used to encompass a dual positive/negative selection cassette within the *hprt* targeting vector. This cassette contains a PGK-neo transcription unit to allow positive selection of G418 resistant transfectants, and a MC1-thymidine kinase (TK) minigene that enables negative selection using gancyclovir, thereby facilitating substitution of the entire cassette by recombination-mediated cassette exchange via flanking heterospecific LoxP and Lox511 sites (Figure 1).

To establish the general applicability of gene targeting in rat ES cells we decided to disrupt the *hprt* gene in cell lines from two rat strains. The Fischer F344 strain was selected as representing an inbred rat that is frequently used in biomedical studies, and was the source of genomic DNA for the homology arms in the targeting vector. The outbred Sprague Dawley (SD) strain was chosen because SD ES cells have previously been shown to contribute to the germ line in chimaeric rats [2]. Cell lines from both strains were derived *de novo* from rat blastocysts using 2i medium and DIAM feeder support cells as described previously [2] (Table 1). Consistent with previous experiments [2] (Buehr and Meek unpublished observations), F344 cell lines grew slower than comparable SD cultures. To stimulate growth of these cultures we tested whether reducing the oxygen tension would aid establishment of F344 cell lines, as this approach had been reported to promote growth of other types of ES cells [17]. Indeed, cell lines from F344 embryos were established more efficiently in 2% than 21% oxygen and expanded more rapidly from embryo outgrowths, indicating that lowering the oxygen tension improves cell proliferation or survival in F344 stem cell cultures (Table S1, Figure S1).

**Table 1:** Rat embryonic stem cell derivation efficiency.

Strain	Oxygen (%)	Outgrowths	Established Lines	Efficiency (%)
SD	21	13	13	100
F344	21	10	7	70
F344	2	11	10	91

Based on their robust growth and karyotype we selected male Fischer (RIF5.2) and SD (RISD10) ES cell lines for the gene targeting experiments (Table 2). The cell lines were transfected with linearised vector DNAs using a standard electroporation protocol previously validated in rat ES cells [2]. After approximately 10 days selection in medium containing the aminoglycoside G418, the antibiotic resistant ES colonies cells were switched to medium containing 6-TG, which eliminates any cells expressing hprt. Untransfected, parental control cells under these conditions were completely eliminated within 3–4 days. In contrast, a small number of G418 resistant colonies, that had been electroporated with the targeting vectors, continued to expand in 6-TG medium, indicating that the endogenous *hprt* gene was inactive in these cells. To establish directly that the *hprt* gene had been disrupted by vector-mediated recombination, we analysed genomic DNA prepared from sample 6-TG resistant clones. PCR amplification with primer combinations using 5′ or 3′ primers located outside the homology arms, produced the expected product sizes of 6.5 kb and 2.7 kb, respectively (Figure 1C). A Southern Blot hybridised with a probe upstream of the 5′ homology arm, confirmed accurate insertion of the targeting vector to delete the region encompassing exons 7–8 in SD and F344 clones (Figure 1D). The efficiency of *hprt* targeting achieved with the rat cells in four independent electroporations, using either the dual selection vector or a simpler neo cassette (Figure S2), was similar to those originally reported for mouse and human ES cells [18], [19], [20] (Table 3). Chromosome counts identified targeted clones with karyotypes close to that of the parental cell lines, indicating that the process of gene targeting and clonal growth under selection is compatible with stable expansion of rat ES cells (Table 2). However, the recovery of aneuploid clones amongst our targeted lines is consistent with an underlying genetic instability in rat ES cell cultures, noted previously [3].

**Table 2:** Karyotype of targeted rat ES cell clones.

Cell Line	Oxygen (%)	Passage	Karyotype (%)#	Plates counted
RIF5.2	2	6	60	50
RIF5.2, 1-B9	2	22	63	38
RIF5.2, 3-B4	2	21	0	36
RISD10	21	10	69	52
RISD10, 6.7, tg13	21	13	68	22
RISD10, 6.7, tg14	21	12	0	20
RISD10, 6.7, tg16	21	13	69	16
RISD10, 2.2, tg14	21	12	64	42

# Percentage of metaphase plates containing euploid chromosome number of 42.

**Table 3:** Comparison of HPRT targeting frequency in ES cells.

Author	Species	Strain	Homology	Expt.	Cell No.	G418^R^	6-TGR¶	Frequency†
Burdon	Rat	F344	4.7 kb	1	2.7×10^6^	58	1	0.4×10^−6^
				2	2.7×10^6^	53	1	0.4×10^−6^
		SD	4.7 kb	3	3.0×10^6^	43	3	1.0×10^−6^
				4*	3.0×10^6^	60	1	0.3×10^−6^
Capecchi [19]	Mouse		9.1 kb	N/A	7.8×10^7^	30000	32	0.4×10^−6^
Smithies [18]	Mouse		1.3 kb	N/A	2.5×10^6^	2000	4	1.6×10^−6^
Thomson [20]	Human		11.9 kb	N/A	1.5×10^7^	350	7	0.5×10^−6^

¶ Selection with G418 first, followed by 6-TG selection.

† Number of targeted cells per number of cells electroporated.

* Electroporation with frt flanked Neo^R^ *hprt* targeting vector.

**Figure 2:**
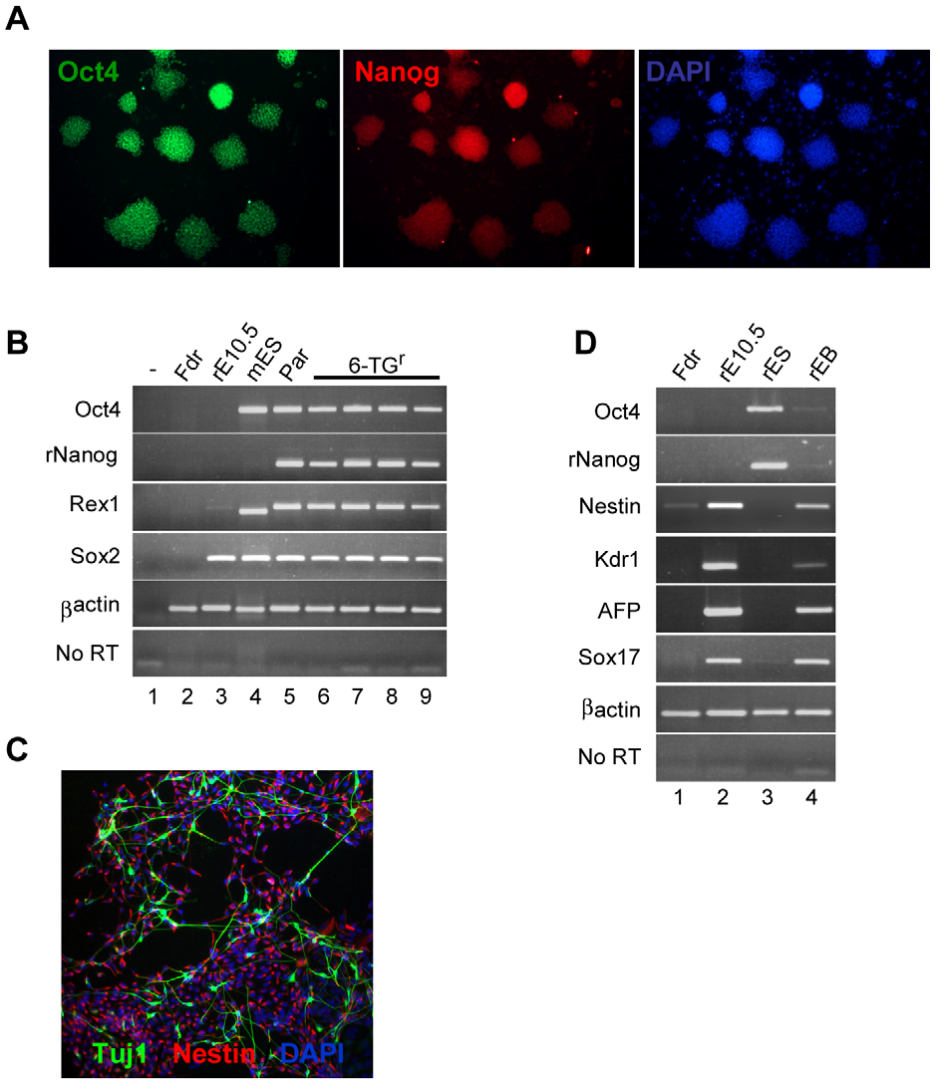
Characterisation of *hprt* -targeted rat F344 embryonic stem cells. (A) Immunohistochemical staining of targeted clone 1-B9 for Oct4 and Nanog (Magnification x100). (B) RT-PCR analysis of (1) Water blank, (2) DIA-M feeder layer, (3) rat E10.5 embryo, (4) E14Tg2a mouse ES cells, (5) RIF5.2 parental rat ES cell line, (6) 6-TG-resistant clone 1-B9, (7) 6-TG-resistant clone 2 F10, (8) 6-TG-resistant clone 3-B4 and (9) 6-TG-resistant clone 3-C10. (C) Immunostaining for Nestin and Tuj1 following 11 day monolayer differentiation protocol of 6-TG-resistant clone 1-B9 (Magnification x100). (D) RT-PCR analysis of (1) DIA-M feeder layer, (2) rat E10.5 embryo, (3) 6-TG-resistant clone 1-B9, (4) Embryoid bodies formed from clone 1-B9.

To establish whether pluripotency was maintained in the targeted clones, we evaluated the stem cell marker profile and *in vitro* differentiation capacity of F344 and SD targeted clones (Figure 2 and Figure S3). This analysis showed that the RNA and protein expression patterns of the stem cell markers Oct4, Nanog, etc was equivalent to that of the parental cell line. Moreover, RT-PCR, and immunohistochemical analysis of cells differentiated either in suspension or in monolayer confirmed that targeted cells were capable of giving rise to cells from all three embryonic germ layers, ectoderm (TuJ, Nestin staining) endoderm (Sox17, AFP) and mesoderm (Flk1).

Based on the data reported here, rat ES cells are readily amenable to gene targeting by homologous recombination using the basic methodology that has proved so effective in mouse ES cells. The efficiency of targeted gene insertion at the rat *hprt* locus was similar to the first published reports for mouse and human ES cells. This demonstrates that rat cells derived and maintained in the 2i growth conditions are readily amenable to genetic modification, and should encourage efforts to achieve routine gene targeting in the rat. The important next steps in achieving this aim will be to refine culture methods in order to maximise ES cell integrity, and to identify stem cell/embryo strain combinations that ensure reliable and efficient transmission of genetic modified cells through the germ line. The availability of *hprt* deficient rat ES cell lines also provides reagents with which to develop reliable, controllable transgene expression in rat ES cells, as well as accelerating the prospect of using large scale genome engineering to humanise specific chromosomal regions in the rat.

## Materials and Methods

### Ethics statement

All animal work conformed to guidelines for animal husbandry according to the UK Home Office, and ethics were approved by the Roslin Institute Animal Ethics Committee. Animals were naturally mated, and sacrificed under schedule 1; procedures that do not require specific Home Office approval.

### Derivation of rat ES cells

Rat blastocysts were harvested at 4.5d.p.c in PB1 containing 10% FCS and the zona pellucida removed with acid Tyrode's solution (Sigma, T1788). The blastocysts were then incubated in 20% anti-rat serum antibody (Sigma, R5256) for 3 hours at 37°C, then rinsed three times in successive drops of PB1 containing 10% FCS. The trophectoderm was lysed at 37°C for 20 minutes in PB1 containing rat serum, collected in-house, at a dilution of 4∶1. The resulting ICMs were isolated using a fine, glass pipette, then plated on a layer of gamma-irradiated DIA-M cells in 2i medium [2]. Cells were allowed to attach and outgrow for approximately five days, then mechanically disaggregated into small clumps using a fine, glass pipette and moved to fresh DIA-M feeders in 2i medium.

### Culture Procedure

Feeder cells were prepared from gamma-irradiated DIA-M mouse fibroblasts that express matrix-associated LIF [21], [22]. ICMs and passaged cell lines were plated on DIA-M feeders in 2i medium [2]. Inhibitors were custom-synthesized by the Division of Signal Transduction Therapy, University of Dundee. Cell lines were routinely passaged by aspirating the colonies into fine, glass pipettes and transferring the resultant disaggregated cells to fresh wells or by dissociation with Cell Dissociation Buffer.

Monolayer neural differentiation was induced by adherent serum-free culture for 11 days using laminin as a substrate for attachment.

### DNA Cloning

A 6.9 kb fragment was amplified from F344 genomic DNA using oligonucleotides flanking Exons 7, 8 and 9 (HPRTintr6for2 - CCTCCCCAATGCCTACAATG and 3′FLKrev3 – CCTTTCCCTGTCCTACACAC). The PCR was performed using Pfu UltraII Fusion HS DNA Polymerase (Stratagene, 600672) under the following conditions; 95°C for 2 minutes, followed by 30 cycles of 94°C for 30 seconds, 60°C for 30 seconds and 68°C for 10 minutes, with a final extension at 68°C for 10 minutes. PCR products were cloned into *Eco*RV digested pBluescript and sequence integrity confirmed by comparing sequences of four individual clones from separate PCR reactions with the sequence for Brown Norway rat (Ensembl, ENSRNOG00000031367).

A 5.4 kb *Bst*BI/*Sac*I fragment was subcloned from the F344 HPRT PCR clone. The 650 bp *Nde*I/*Eco*RV fragment containing exons 7 and 8 was removed and replaced with either a double selection cassette consisting of PGK*neo* and MC1*tk*, flanked by inverted heterospecific *lox* sites, *loxP* and *lox511*, or an frt-flanked PGKneo cassette.

### Electroporation

The targeting vector containing the *neo*/*tk* double selection cassette was linearised with *Ahd*I. The targeting vector containing the *neo* single selection cassette was linearised with *Xho*I. Approximately 3×10^6^ rat ES cells were electroporated in PBS containing 50 µg linearised HPRT targeting vector using the Bio-Rad Genepulser apparatus (0.8 kV, 3 µF). Electroporated cells were plated into 10 cm^2^ wells containing 2i medium and feeder support cells. G418 (150 µg/ml) selection was added 48 h post-electroporation and the number of G418-resistant colonies counted 9–10 days post-electroporation. 6-TG selection (5 µM) was applied at either day 9, or following picking and replica-plating of individual G418-resistant clones. To reduce HPRT cross-feeding, 6-TG resistant rat ES cells were selected on HPRT-deficient SNL feeder cells.

### Genomic PCR screening

200 ng of genomic DNA was amplified using oligonucleotides designed to identify the 5′ targeting event (HPRT5′FLKfor - GGTAGTAACAAGTGGTGGAC and HPRT3662R – CCACTTTCGCTGATGACAC) and 3′ targeting event (MC1*tk*for – GGGGAATGGTTTATGGTTCG and HPRT3′FLKrev – CAAATGCAGGGAACGACACC). The PCR was performed using Pfu UltraII Fusion HS DNA Polymerase (Stratagene, 600672) under the following conditions; 95°C for 2 minutes, followed by 35 cycles of 94°C for 30 seconds, 60°C for 30 seconds and 68°C for 10 minutes, with a final extension at 68°C for 10 minutes. Products were visualised with ethidium bromide on a 0.8% TBE agarose gel. Expected sizes for 5′ screening are 3.5 kb for wildtype and 6.5 kb for targeted. Expected sizes for 3′ screening are no product for wildtype and 2.7 kb for targeted.

### Southern Blot

Eight micrograms of genomic DNA were digested with 200 units of appropriate enzyme at 37°C for 30 hours. The resulting DNA fragments were resolved on a 0.7% TAE agarose gel overnight at 25 V. The DNA fragments were UV-nicked prior to transfer to Hybond N+ Nylon membrane (GE Healthcare, RPN203B) as described in the manufacturer's instructions. Following transfer, the DNA was UV cross-linked on to the membrane. Probes were prepared by PCR amplification of HPRT sequence flanking the 5′ and 3′ homology arms (Intr6F2 – CCTCCCCAATGCCTACAATG and HPRT289R – GAAAAAGGAAGCAAGTGTGG, and HPRT6125F – GTGCTGTTTTCCTCATGGGC and HPRT6373R – GCTACCTTCTGGCTTTGTTAG for 5′ and 3′ probes respectively).

25 ng of probe DNA was radioactively labelled with α–dCTP P^32^ using High Prime (Roche, 11 585 592 001), then hybridised to the membrane overnight at 65°C in Church solution containing 10 µg/ml sonicated Herring Sperm DNA and 10 µg/ml tRNA. Non-specific binding was removed by washing in 2xSSC/0.1% SDS at 65°C.

### Immunohistochemistry

Cells were fixed in 4% paraformaldehyde (10 minutes at room temperature), washed four times with PBST (PBS, 0.03% TritonX-100), then incubated with blocking solution (PBST, 3% goat serum, 1% BSA) for one hour at room temperature. Primary antibodies were diluted in blocking solution and applied overnight at 4°C, followed by four washes with PBST. Secondary antibodies were diluted 1∶1000 in blocking solution and applied for one hour, at room temperature in the dark. The cells were washed four times with PBST, with the final wash containing 10 µg/ml DAPI. The antibodies used were Oct4 (C-10) primary antibody at 1∶200 (Santa Cruz, sc5279) with goat-anti-mouse IgG2b secondary antibody, Nanog primary antibody at 1∶200 (Abcam, Ab21603) with goat-anti-rabbit IgG secondary antibody, βIII-tubulin primary antibody at 1∶500 (Covance, mms-435P) with goat-anti-mouse IgG2a secondary antibody, and Nestin primary antibody at 1∶20 (Developmental Studies Hybridoma Bank, Rat-401) with goat-anti-mouse IgG1 secondary antibody.

### RT-PCR

RNA was purified from around 50 colonies using RNeasy Mini Kit

(Qiagen) and cDNA subsequently generated by Oligo-dT priming using

SuperScript First-Strand Synthesis System (Invitrogen). The resultant cDNA was diluted to 200 ng/µl, and 2 µl PCR amplified on a PTC-200 thermocycler (MJ Research) using Taq DNA Polymerase (Invitrogen) for 30 cycles. Details of the annealing temperatures, oligonucleotide sequences and product sizes are listed in Table S2. PCR products were resolved on a 2% agarose gel and visualised with ethidium bromide.

## Supporting Information

Figure S1 Effect of oxygen concentration on rat embryonic stem cell growth. (A) Brightfield images of two F344 cell lines, 23 days post-derivation, maintained in 2% or 21% oxygen (magnification x100). (B) Growth rate of six F344 lines, from inner cell mass isolation to passage four, grown in either 2% or 21% oxygen.
(0.47 MB TIF)

Figure S2 Targeting of the HPRT gene in Sprague Dawley rat embryonic stem cells with a PGK/neo targeting vector. (A) Targeting diagram as described in Figure 1A, except that an frt flanked PGK/neo cassette was used to replace exons 7 and 8. (B) Confirmation of targeted integration by Southern blot analysis of XbaI digested genomic DNA from (1) RISD10 parental cell line, (2) RISD10-derived 6-TG-resistant clone 2.2.14 using 5′ probe shown in (A). (C) Brightfield image of RISD10 targeted cell line 2.2.14 (Magnification x100).
(1.48 MB TIF)

Figure S3 Characterisation of HPRT targeted Sprague Dawley rat embryonic stem cells. (A) Immunohistochemical staining of targeted clones 2.2.14 and 6.7.16 for Oct4 and Nanog (Magnification x100). (B) RT-PCR analysis of (1) Water blank, (2) DIA-M feeder layer, (3) rat E10.5 embryo, (4) rat ES cell parental line, (5) 6-TG-resistant clone 2.2.14, (6) 6-TG-resistant clone 6.7.16. (C) Immunostaining for Nestin and Tuj1 following 11 day monolayer differentiation protocol of 6-TG-resistant clones 2.2.14 and 6.7.16 (Magnification x100). (D) RT-PCR analysis of (1) Water blank, (2) DIA-M feeder layer, (3) rat E10.5 embryo, (4) 6-TG-resistant clone 2.2.14, (5) Embryoid bodies formed from clone 2.2.14, (6) 6-TG-resistant clone 6.7.16 and (7) Embryoid bodies formed from clone 6.7.16.
(10.38 MB TIF)

Table S1 Effect of Oxygen levels on rat ES cell growth.
(0.03 MB DOC)

Table S2 RT-PCR oligonucleotides.
(0.05 MB DOC)
