# Supplementary figures and images for: Efficient Gene Targeting by Homologous Recombination in Rat Embryonic Stem Cells
Source: PLoS One. 2010 Dec 3;5(12):e14225. doi: 10.1371/journal.pone.0014225 (PMC2997056; doi:10.1371/journal.pone.0014225)

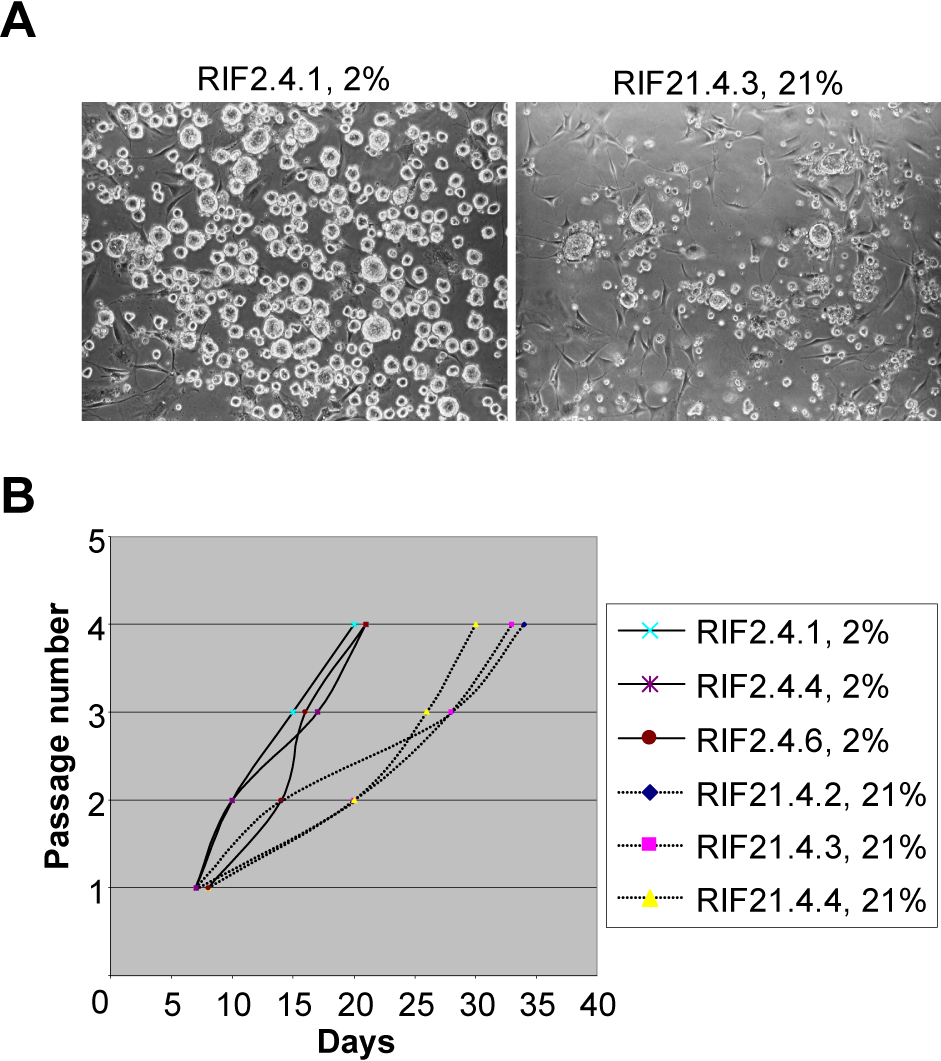

Supplement: Figure S1 — Effect of oxygen concentration on rat embryonic stem cell growth. (A) Brightfield images of two F344 cell lines, 23 days post-derivation, maintained in 2% or 21% oxygen (magnification x100). (B) Growth rate of six F344 lines, from inner cell mass isolation to passage four, grown in either 2% or 21% oxygen. (0.47 MB TIF) [file pone.0014225.s001.tif]

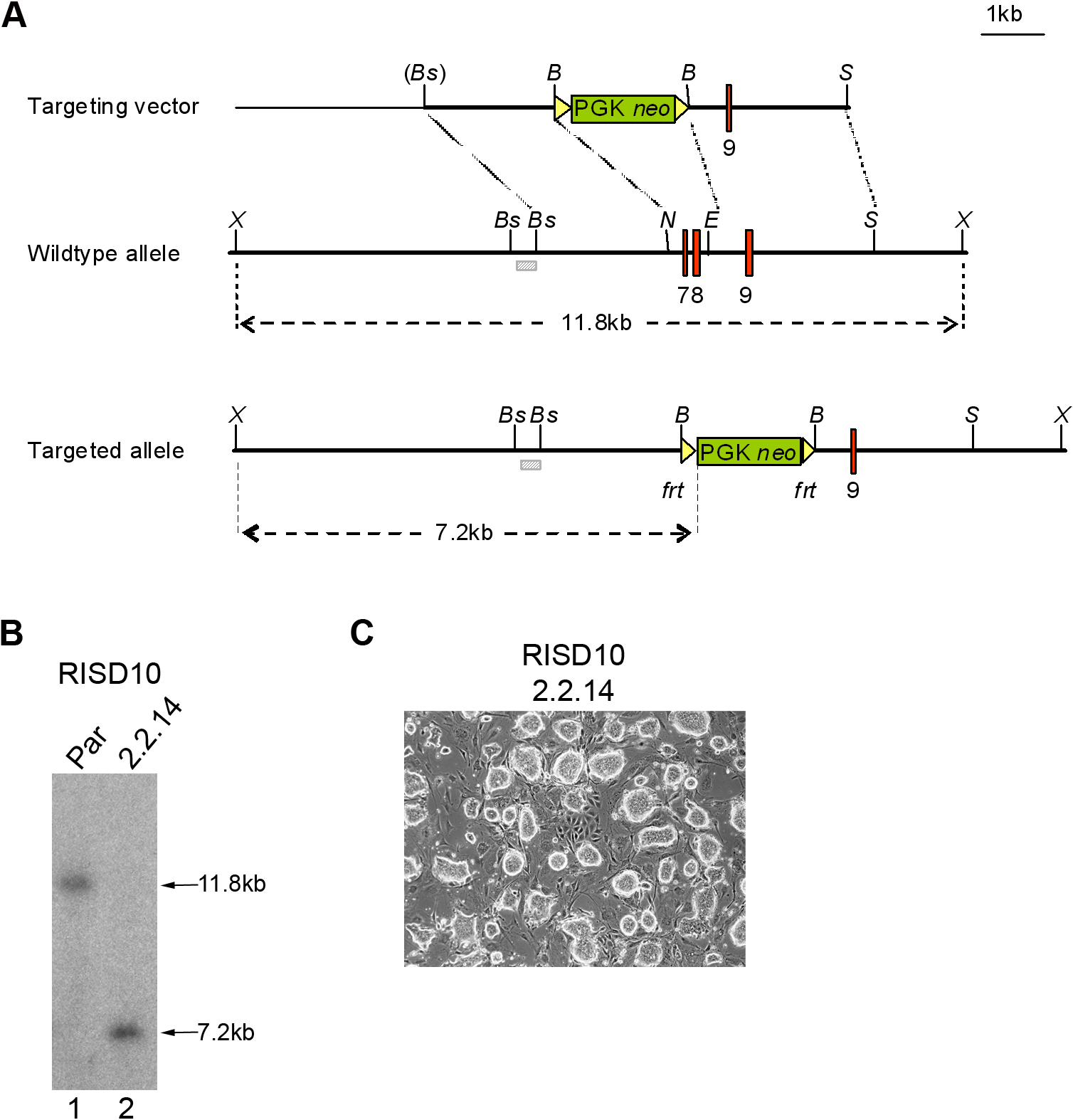

Supplement: Figure S2 — Targeting of the HPRT gene in Sprague Dawley rat embryonic stem cells with a PGK/neo targeting vector. (A) Targeting diagram as described in Figure 1A, except that an frt flanked PGK/neo cassette was used to replace exons 7 and 8. (B) Confirmation of targeted integration by Southern blot analysis of XbaI digested genomic DNA from (1) RISD10 parental cell line, (2) RISD10-derived 6-TG-resistant clone 2.2.14 using 5′ probe shown in (A). (C) Brightfield image of RISD10 targeted cell line 2.2.14 (Magnification x100). (1.48 MB TIF) [file pone.0014225.s002.tif]

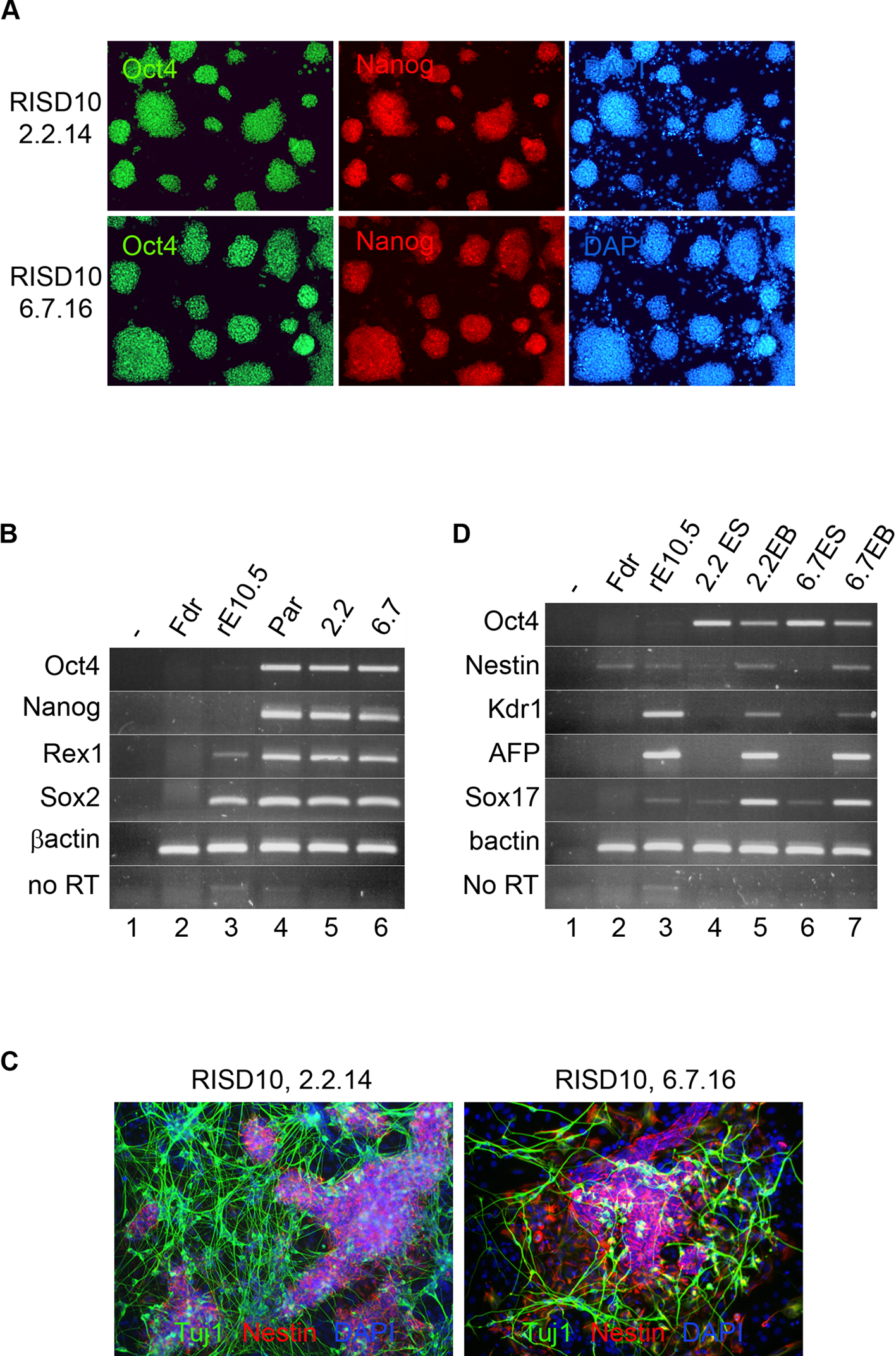

Supplement: Figure S3 — Characterisation of HPRT targeted Sprague Dawley rat embryonic stem cells. (A) Immunohistochemical staining of targeted clones 2.2.14 and 6.7.16 for Oct4 and Nanog (Magnification x100). (B) RT-PCR analysis of (1) Water blank, (2) DIA-M feeder layer, (3) rat E10.5 embryo, (4) rat ES cell parental line, (5) 6-TG-resistant clone 2.2.14, (6) 6-TG-resistant clone 6.7.16. (C) Immunostaining for Nestin and Tuj1 following 11 day monolayer differentiation protocol of 6-TG-resistant clones 2.2.14 and 6.7.16 (Magnification x100). (D) RT-PCR analysis of (1) Water blank, (2) DIA-M feeder layer, (3) rat E10.5 embryo, (4) 6-TG-resistant clone 2.2.14, (5) Embryoid bodies formed from clone 2.2.14, (6) 6-TG-resistant clone 6.7.16 and (7) Embryoid bodies formed from clone 6.7.16. (10.38 MB TIF) [file pone.0014225.s003.tif]
