# Supplementary material for: Efficient Gene Targeting by Homologous Recombination in Rat Embryonic Stem Cells
Source: PLoS One. 2010 Dec 3;5(12):e14225. doi: 10.1371/journal.pone.0014225 (PMC2997056; doi:10.1371/journal.pone.0014225)
Supplement: Table S1 — Effect of Oxygen levels on rat ES cell growth. (0.03 MB DOC) [file pone.0014225.s004.doc]

**Table S1.** Effect of Oxygen levels on rat ES cell growth.

| **Cell Line** | **Oxygen (%)** | **Days to P4** |
| --- | --- | --- |
| RIF21.4.2 | 21 | 34 |
| RIF21.4.3 | 21 | 33 |
| RIF21.4.4 | 21 | 30 |
| RIF2.4.1 | 2 | 20 |
| RIF2.4.4 | 2 | 21 |
| RIF2.4.6 | 2 | 21 |
