# Supplementary material for: Efficient Gene Targeting by Homologous Recombination in Rat Embryonic Stem Cells
Source: PLoS One. 2010 Dec 3;5(12):e14225. doi: 10.1371/journal.pone.0014225 (PMC2997056; doi:10.1371/journal.pone.0014225)
Supplement: Table S2 — RT-PCR oligonucleotides. (0.05 MB DOC) [file pone.0014225.s005.doc]

**Table S2.** RT-PCR oligonucleotides.

| **mRNA** | **Rat oligonucleotide sequence** | **Product size (bp)** | **Ann. Temp. (^o^C)** |
| --- | --- | --- | --- |
| Afp | For - GCCAAAGTGGAGTGGAAAGA | 249 | 50 |
|  | Rev - TGTTGTCAGCTTTGCAGCAT |  |  |
| β-actin | For - CATGGCATTGTGATGGACT | 427 | 58 |
|  | Rev - ACGGATGTCAACGTCACACT |  |  |
| Fgf4 | For - CGGGGTGTGGTGAGCATCTTC | 202 | 50 |
|  | Rev - CCTTCTTGGTCCGCCCGTTC |  |  |
| Kdr | For - ATACACCTGCACAGCGTACAG | 271 | 50 |
|  | Rev - TCCCGCATCTCTTTCACTCAC |  |  |
| Nanog | For - GCCCTGAGAAGAAAGAAGAG | 356 | 50 |
|  | Rev - CGTACTGCCCCATACTGGAA |  |  |
| Nestin | For -AGAGAAGCGCTGGAACAGAG | 234 | 50 |
|  | Rev - AGGTGTCTGCAACCGAGAGT |  |  |
| Oct4 | For - GGGATGGCATACTGTGGAC | 412 | 50 |
|  | Rev - CTTCCTCCACCCACTTCTC |  |  |
| Rex1 | For - TTCTTGCCAGGTTCTGGAAGC | 297 | 50 |
|  | Rev - TTTCCCACACTCTGCACACAC |  |  |
| Sox2 | For - GGCGGCAACCAGAAGAACAG | 414 | 50 |
|  | Rev - GTTGCTCCAGCCGTTCATGTG |  |  |
| Sox17 | For - AGGAGAGGTGGTGGCGAGTAG | 268 | 50 |
|  | Rev - GTTGGGATGGTCCTGCATGTG |  |  |
